# Maternal nicotine exposure induces congenital heart defects in the offspring of mice

**DOI:** 10.1111/jcmm.17328

**Published:** 2022-05-06

**Authors:** Elizabeth R. Greco, Anish Engineer, Tana Saiyin, Xiangru Lu, MengQi Zhang, Douglas L. Jones, Qingping Feng

**Affiliations:** ^1^ Department of Physiology and Pharmacology London Ontario Canada; ^2^ Department of Medicine Schulich School of Medicine and Dentistry Western University London Ontario Canada

**Keywords:** congenital heart defects, embryonic heart development, maternal nicotine exposure, nicotine replacement therapy

## Abstract

Maternal cigarette smoking is a risk factor for congenital heart defects (CHDs). Nicotine replacement therapies are often offered to pregnant women following failed attempts of smoking cessation. However, the impact of nicotine on embryonic heart development is not well understood. In the present study, the effects of maternal nicotine exposure (MNE) during pregnancy on foetal heart morphogenesis were studied. Adult female mice were treated with nicotine using subcutaneous osmotic pumps at 0.75 or 1.5 mg/kg/day and subsequently bred with male mice. Our results show that MNE dose‐dependently increased CHDs in foetal mice. CHDs included atrial and ventricular septal defects, double outlet right ventricle, unguarded tricuspid orifice, hypoplastic left ventricle, thickened aortic and pulmonary valves, and ventricular hypertrophy. MNE also significantly reduced coronary artery size and vessel abundance in foetal hearts. Moreover, MNE resulted in higher levels of oxidative stress and altered the expression of key cardiogenic regulators in the developing heart. Nicotine exposure reduced epicardial‐to‐mesenchymal transition in foetal hearts. In conclusion, MNE induces CHDs and coronary artery malformation in mice. These findings provide insight into the adverse outcomes of foetuses by MNE during pregnancy.

## 1 INTRODUCTION

Congenital heart defects (CHDs) are the most common birth defect occurring in about 1% of live births.^1^ While genetic factors are identified in about 15% of cases, the cause of most CHDs (85%) remains unknown and may include environmental factors.^2^ Maternal cigarette smoking is an environmental risk factor for CHDs as well as other congenital anomalies.^3^, ^4^ The human adverse effects of cigarette smoking have been examined extensively and attributed to many toxins found in the tobacco smoke. In the United States, United Kingdom and Australia, approximately 12%–15% of women still smoke during pregnancy.^5^ Annually in Canada, 75,000 newborns are affected by maternal smoking, and in Northern Canada, as high as 59% of the newborns are impacted.^6^ Although many pregnant women may appreciate the danger of smoking, cessation is challenging because nicotine is the highly addictive and only 46%–54% abstain from smoking during pregnancy.^7^, ^8^

To reduce the prevalence of maternal smoking, pharmacotherapies such as nicotine replacement therapies (NRTs) are often offered to pregnant women following failed attempts at smoking cessation.^9^, ^10^ Another popular method for smoking cessation is nicotine containing electronic cigarettes (e‐cigarettes). In young adults of reproductive age (20–28), the use of e‐cigarettes is rapidly increasing.^11^, ^12^ Although NRTs and e‐cigarettes contain nicotine, they are perceived to be less harmful than cigarette smoking since they do not contain additional toxins found in tobacco smoke. The relationship between NRTs/e‐cigarettes and congenital anomalies is poorly understood. Additional studies are required to elucidate the nicotine's effects on foetal development.^13^ Currently available clinical evidence on the foetal safety of maternal use of NRTs during pregnancy is discordant.^14^, ^15^, ^16^, ^17^ It remains unclear whether these replacements are safe for the foetuses.

Maternal nicotine exposure (MNE) during pregnancies is more likely to have adverse outcomes.^18^ In animal studies, MNE adversely affects various organs during foetal development, including exacerbation of fibrosis of lungs and kidney, alterations in brain structure and function, mitochondrial dysfunction in the pancreas and impairment of structure and function of the placenta.^19^ In foetal hearts, nicotine delays the development of the sinoatrial node and induces cardiac arrhythmia.^20^, ^21^ Further, nicotine reduces blood oxygenation and increases blood pressure in foetuses.^18^

Increases in oxidative stress may contribute to these detrimental effects of foetal nicotine exposure.^22^ Reactive oxygen species (ROS) such as superoxide and hydrogen peroxide mediate many fundamental cellular processes critical to embryonic development including cell differentiation, proliferation, migration and programmed cell death.^23^, ^24^ Elevated ROS levels induce oxidative stress, which alters genetic expression and disrupts developmental processes. In foetal hearts, oxidative stress contributes to the pathogenesis of CHDs and coronary artery malformation.^25^, ^26^ To our knowledge, there is no report on the direct effects of MNE on the pathogenesis of CHDs. We hypothesized that MNE during pregnancy would impair foetal heart development, resulting in CHDs and hypoplastic coronary arteries. We tested this hypothesis using subcutaneous osmotic pumps administering clinically relevant doses of nicotine to female mice during gestation.

## 2 MATERIALS AND METHODS

### 2.1 Animals

This study used mice in accordance with the Guide to Care and Use of Animals of the Canadian Council of Animal Care and was approved by the Animal Care Committee at Western University, Canada. C57Bl/6 mice were purchased from Charles River Laboratories, Canada. All efforts were made to minimize the number of animals used and to minimize their suffering. Animals were housed in a 12 h light/dark cycle and had access to standard chow and water. Females at 8–10 weeks of age had osmotic pumps (Alzet #2004,) implanted subcutaneously, releasing nicotine at 0.75 or 1.5 mg/kg/day. These doses mimic a light smoker, of 1–10 cigarettes per day.^27^, ^28^, ^29^ Control mice did not receive pump implantation. Fourteen days after implantation of the pump, the females were bred overnight in cages with healthy males, then returned to their original cage in the morning. The presence of a vaginal plug indicated embryonic day (E) 0.5. Females that had an unsuccessful pregnancy following the presence of the plug were bred again with males. After 2 unsuccessful pregnancies, females were sacrificed and not included in analysis.

Embryos from pregnant mice with or without maternal nicotine exposure (MNE) were collected at E10.5 to E18.5 via caesarean section under ketamine (50 mg/kg, IP) and xylazine (10 mg/kg, IP) anaesthesia. Dams were sacrificed by cervical dislocation after embryos were collected. Embryonic/foetal hearts were harvested at E10.5 to analyse mRNA and oxidative stress during embryonic development; at E12.5 to analyse epicardial EMT potential, and at E18.5 to analyse morphology and mRNA. For mRNA analysis, hearts were flash frozen in liquid nitrogen and stored at −80°C freezer.

### 2.2 Osmotic mini pumps

Alzet (#2004) 28‐day osmotic minipumps were prepared 24 h prior to implantation. The online Alzet pump calculator was used to determine the dose with which to fill the pump with nicotine ((‐)‐nicotine N3876, Sigma‐Aldrich) solution according to the weight of the animal. An intramuscular injection of a mixture of ketamine (25 mg/ml), xylazine (2.5 mg/ml) and atropine (30 µg/ml) was used to anaesthetize the mice for surgical pump implantation. A small incision was made slightly posterior to the scapulae, and the pump was implanted. The incision was closed with silk sutures, with the mice monitored throughout recovery. To relieve pain, buprenorphine (0.05 mg/kg, s.c., q8h) was used for 2 days postsurgery.

### 2.3 Histological Analysis of CHD

Neonates (P0) and foetuses at E17.5 and E18.5 were collected for histological analysis. To collect foetuses, pregnant mice were anaesthetized with an intramuscular injection anaesthetic mixture (as above), and a caesarean section was performed.^30^ The upper torso of foetal and neonatal mice were isolated and immediately fixed in 4% paraformaldehyde for 18 h at 4 °C, then dehydrated in ethanol and embedded in paraffin. Samples were serially sectioned at 5 μm thickness using a Leica RM2255 microtome. Transverse sections started at the level of the thymus (just above the aortic arch) and continued until after the apex of the heart. Heart sections were stained with haematoxylin/eosin (H/E) to diagnose CHDs during blinded examination under a light microscope (Zeiss Observer D1,). All quantifications of histological images were performed using ZEN microscope software (Zeiss). The diameters of the proximal left and right coronary arteries were measured at 50 μm from the left coronary artery ostium and on a section 250 μm below the left coronary artery ostium where the right coronary artery was the most prominent, respectively.

### 2.4 Immunohistochemistry

E18.5 foetal heart sections were immunostained using anti‐α‐smooth muscle actin primary antibody (1:3000 dilution, Abcam,) to visualize coronary arteries.^31^ Sections were blocked in a solution of 0.3% H_2_O_2_ diluted in phosphate buffered saline (PBS) prior to incubation in secondary antibody. The primary antibody was left on overnight at room temperature in a humidity chamber. The secondary antibody, biotinylated goat anti‐rabbit IgG (1:500) (Vector laboratories), was subsequently left on sections for 1 h at room temperature in a humidity chamber. All antibodies were diluted in tris‐buffered saline with Tween‐20 (TBST). Sections were then incubated in ABC reagent (1:200 in PBS, Vector Laboratories) for 40 min and visualized using 3–3’ di‐aminobenzidine tetrahydrochloride (DAB, Sigma‐Aldrich,) and H_2_O_2_. All immunostained sections were counterstained with Mayer's Hematoxylin (Thermo Fisher Scientific).

### 2.5 Three‐dimensional reconstruction of coronary arteries

E18.5 serial heart sections 25 μm apart were immunostained with α‐smooth muscle actin to visualize coronary arteries. Images were taken using a Zeiss Observer D1 microscope and processed with the AMIRA^®^ software (Template Graphics Software) to reconstruct three‐dimensional (3D) model representations. The compact myocardium, and the right and left ventricles or coronary arteries were labelled manually in each section. The volume of the labelled components was calculated, and a ratio of coronary artery to myocardial volume was obtained.^31^

### 2.6 Real‐time PCR analysis

E10.5 and E18.5 hearts were isolated and snap frozen in liquid nitrogen. Total RNA was extracted from ventricular tissues of E10.5 and E18.5 hearts using the TRIzol reagent method. Reverse transcription with M‐MLV reverse transcriptase (Invitrogen, Canada) was performed using 0.2 µg of total RNA in a reaction mixture totaling 20 µl. Following reverse transcription, real‐time quantitative PCR amplification was performed using EvaGreen qPCR MasterMix (Abm, Vancouver, Canada). Primers were designed to amplify the genes *Nkx2*.*5*, *Gata4*, *Bmp10*, *eNOS*, *Notch1*, *CyclinD1*, *Hif*‐*1α*, *bFGF*, *Tbx5*, *Tbx18*, *Snail1*, *Slug*, *ALDH1a2*, *TGF*‐β*1*, *PKCi*, *β*‐*MHC*, *BNP*, *SX* and 28S (Table S1). An Eppendorf MasterCycler Realplex (Eppendorf) was used to amplify qPCR mixtures for 35 cycles at temperatures set in accordance with the primer melting temperatures. The Ct values of target genes were normalized to 28S ribosomal RNA using a comparative Ct method.

### 2.7 Analysis of superoxide, lipid peroxidation and cell proliferation

Frozen E10.5 hearts from both control and MNE groups were sectioned into 8 μm sections using a cryostat (CM1950, Leica). Dihydroethidine (DHE, 2 μM), a molecular probe for superoxide, was used to measure relative levels of ROS by quantifying fluorescence densitometry in the absence or presence of 100 units/ml of superoxide dismutase (SOD). Fluorescence was imaged using a microscope (Observer D1, Zeiss). Three to 5 images were taken from 5 different sections per heart sample at a fixed exposure time. AxioVision Microsoft software (Observer D1, Zeiss) was used to quantify the signal intensity per area of myocardium.

Lipid peroxidation, another indicator of cellular oxidative stress, was measured in E10.5 frozen heart sections. Slides were incubated with anti‐4‐hydroxynonenal (4‐HNE) primary antibody (1:300, Abm, Vancouver, Canada) overnight at room temperature. A fluorescent‐labelled donkey anti‐goat secondary antibody (1:1000, LI‐COR Biosciences) used to visualize lipid peroxidation, was incubated for 1 h and followed by Hoechst stain (1:1000) to label the nuclei. Images were taken at a constant exposure in heart sections of all groups using a fluorescence microscope (Observer D1, Zeiss). The fluorescence intensity was quantified using the AxioVision program (Zeiss).

Anti‐phosphorylated histone H3 (pHH3) antibody (1:1000, Abcam) was used for detection of phosphorylated histone H3, a marker specific for cells undergoing mitosis, in E10.5 frozen heart sections. The primary antibody was left on for 2 h at room temperature in a humidity chamber. Sections were then incubated for 1 h in secondary antibody (1:1000, Cy™3‐conjugated AffiniPure Goat Anti‐Rabbit IgG, Jackson ImmunoResearch Laboratories), followed by Hoechst stain (1:1000) to label nuclei. The number of proliferating cells were quantified from the fluorescence signals.

### 2.8 Epicardial EMT assay

To determine the effect of nicotine on epicardial epithelial‐to‐mesenchymal transition (EMT), ventricles of E12.5 embryos were harvested, cut into smaller fragments, and plated on the hydrated collagen gel. M199 medium (Sigma‐Aldrich) was then added to the culture either with or without nicotine (100 ng/ml, (‐)‐nicotine N3876 Sigma‐Aldrich). After 3 days, images were captured using a phase contrast microscope (Observer D1, Zeiss), and the number of spindle‐shaped cells which had grown outward from the explanted ventricles were quantified as previously described.^26^, ^31^

### 2.9 *In utero* Echocardiography

Directly prior to pup collection, foetal heart function was measured at E18.5 using ultrasound imaging (Vevo 2100) with an MS 750 transducer (VisualSonics).^32^ Maternal mice were pre‐anaesthetized in a chamber, using 3.0% isoflurane, and subsequently secured in the supine position on a heated dock (temperature 37°C) with their noses in a cone used to deliver 0.5–1.5% isoflurane (for anaesthesia maintenance). An incision was made on the lower abdomen to expose the embryotic sacs. M‐mode echocardiography images of the foetal hearts were recorded in the short‐axis view. The end‐diastolic left ventricular internal diameter and end‐systolic left ventricular internal diameter were measured. Ejection fraction and fractional shortening were calculated. Mothers were then anaesthetized with the same mixture as above, and removed from isoflurane for pup collection.

### 2.10 Statistical analysis

Data are presented as means ± SEM. Statistical analysis was performed between control and MNE using unpaired Student's *t*‐test (GraphPad Prism, version 5.0). A Chi‐square or Fisher's exact test was used to analyse the incidence of CHDs and coronary artery malformation. Two‐way analysis of variance (ANOVA) followed by Tukey's multiple comparisons test were used for the analysis of sex differences in the CHD incidence and the DHE data. A *p* value less than 0.05 was considered statistically significant.

## 3 RESULTS

### 3.1 Effects of MNE on foetal weight, placental weight, fertility rate and cardiac function

**FIGURE 1:**
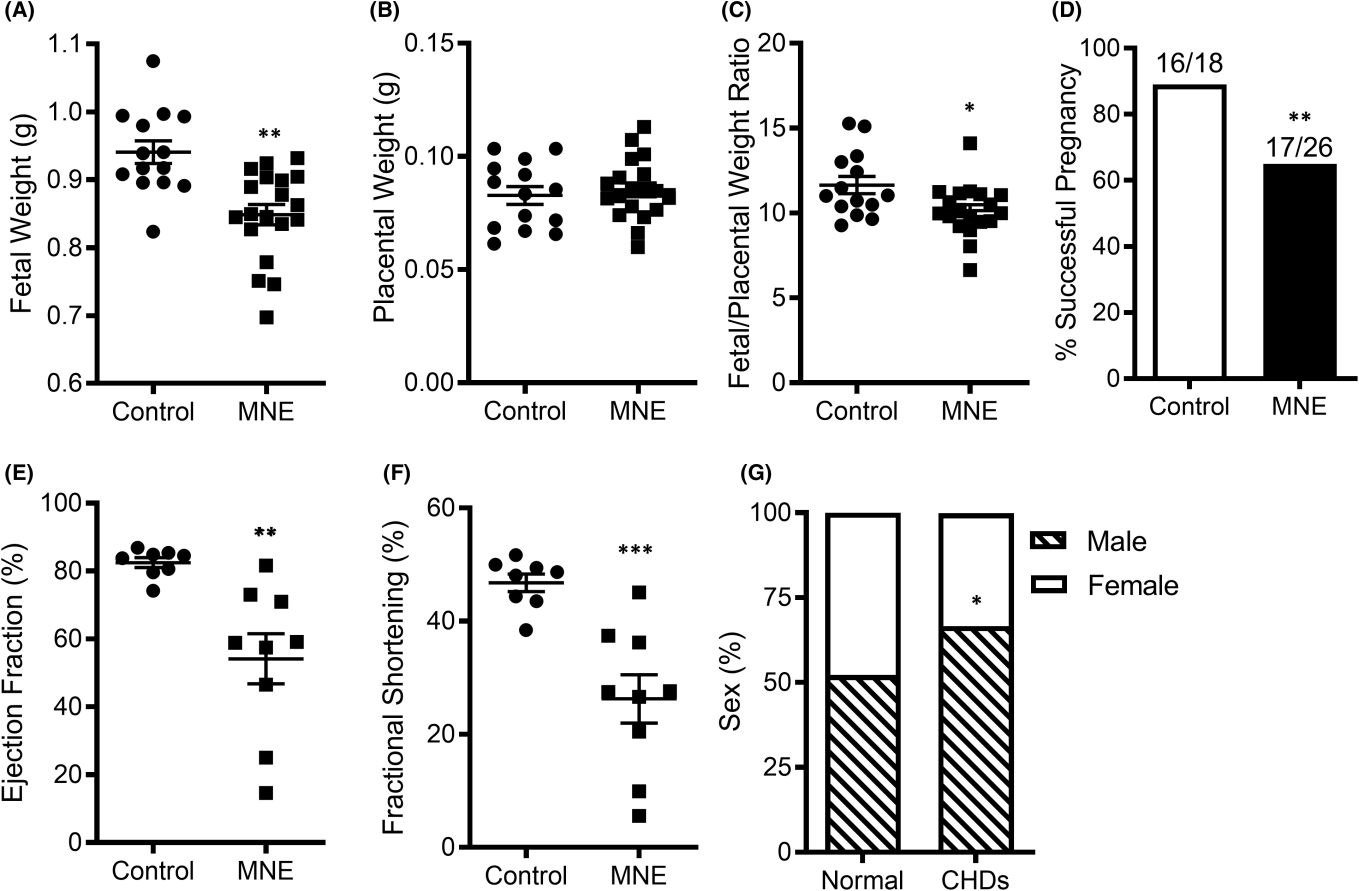
Effects of MNE (1.5 mg/kg/day) on foetal body weight, placental weight, pregnancy rate, cardiac function and CHD sex ratio. Foetal weight (A), placental weight (B) and foetal/placental weight ratio (C) at E17.5 in control (*n* = 14) and nicotine (*n* = 29) groups. (D) Fertility rate (number of successful pregnancies to total vaginal plugs). Left ventricular ejection fraction (E) and fractional shortening (F) were determined using echocardiography *in utero* at E18.5. (G) Percent of male and female foetuses with or without a CHD. An unpaired Student's t‐test (A‐C, E and F) or chi‐squared test (D and G) was performed for statistical analysis. *p < 0.05, **p < 0.01 vs. control

Foetuses and placenta were weighed at collection. While the weight of placenta was similar between control and MNE (1.5 mg/kg/day) groups (*p*=n.s), foetal weights and foetal‐to‐placental weight ratios were significantly lower with MNE (*p* < 0.05, Figure 1A–C). The rate of successful pregnancy (the per cent of vaginal plugs that lead to a successful pregnancy) was lower in MNE (1.5 mg/kg/day) in comparison with controls (*p* < 0.01, Figure 1D). The per cent of absorbed pups had a higher trend in the MNE group (8.1%) than the control group (2.9%, *p* = 0.0548 by Fisher's exact test). Cardiac function of foetuses was assessed by echocardiography *in utero* at E18.5. The ejection fraction and fractional shortening of the left ventricle (LV) were lower in the MNE group in comparison with control foetuses (*p* < 0.01, Figure 1E–F), indicating reduced cardiac function in nicotine‐exposed foetuses. The sex of foetuses was identified by the Y chromosome specific gene using PCR analysis. Our data showed that while the male to female ratio was about 50% in normal foetuses, CHDs were more prevalent in males compared to females (67% vs. 33%, *p* < 0.05, Figure 1G).

### 3.2 MNE induces congenital heart defects in mice

**FIGURE 2:**
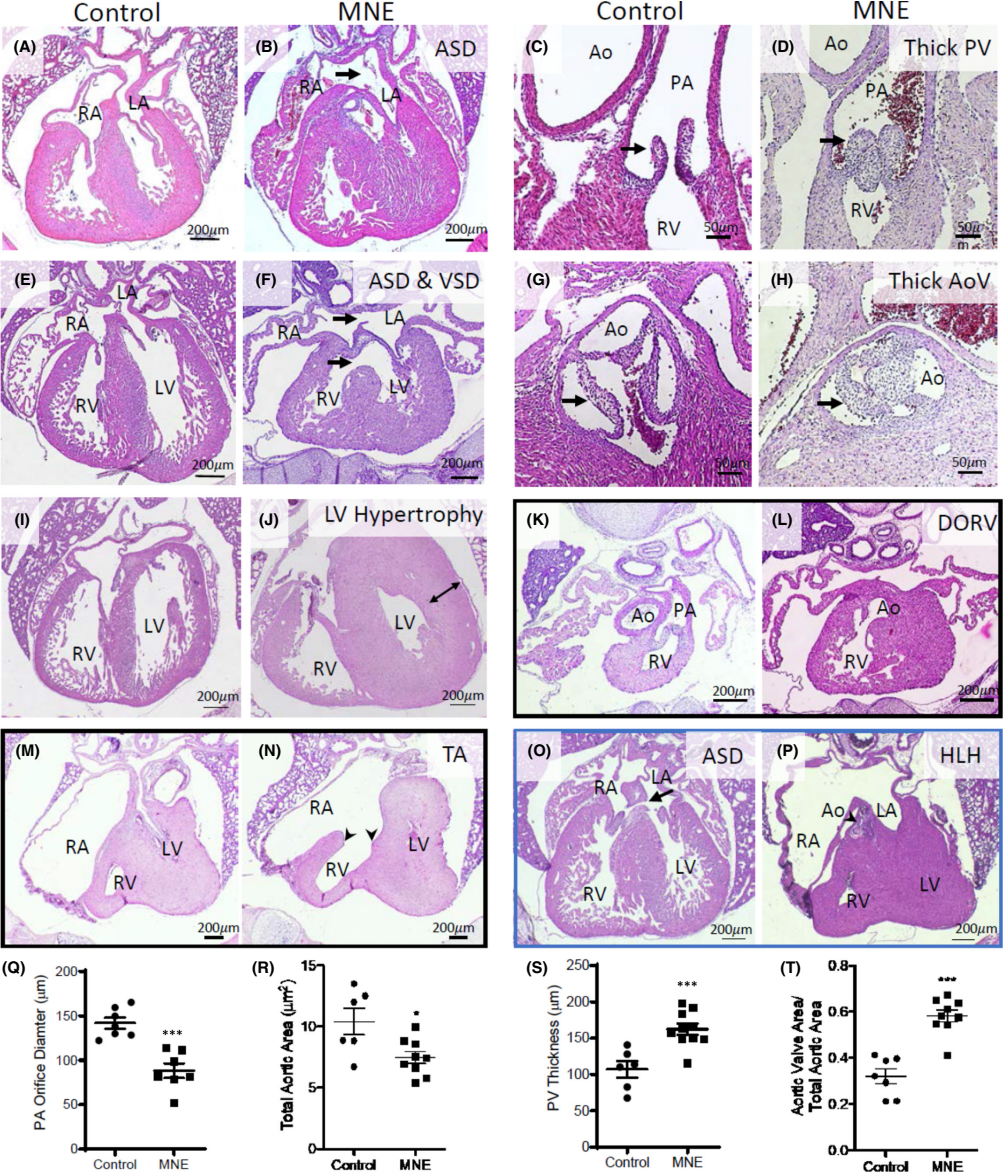
Congenital heart defects (CHDs) in MNE offspring. A, C, E, G and I are normal morphology of heart structures in control offspring. MNE (1.5 mg/kg/day) induced a spectrum of defects including (B) ostium secundum ASD, (D) thickened pulmonary valves, (F) ostium secundum ASD and perimembranous septum VSD, (H) thickened aortic valves, (J) left ventricle hypertrophy, (K, L) double outlet right ventricle (DORV) in which both pulmonary artery (K) and aorta (L) were connected to the RV (two different sections 350 μm apart from the same heart) and (M, N) unguarded tricuspid orifice (absence of tricuspid valve, arrow heads) with no trabeculation of RV (two different sections 300 μm apart from the same heart). MNE (0.75 mg/kg/day) induced (O) ostium primum ASD and (P) hypoplastic left heart (HLH) with an ostium secundum ASD, aortic stenosis and hypoplastic LV. Q‐T show effects of MNE (1.5 mg/kg/day) on pulmonary orifice diameter (Q), total aortic orifice area (R), total pulmonary valve thickness (S), and aortic valve area to total aortic orifice area (T). Ao, aorta; PA, pulmonary artery; RA, right atrium; LA, left atrium; LV, left ventricle; RV, right ventricle. All images are from E18.5 hearts. *p < 0.05, **p < 0.01, ***p < 0.001 vs. untreated control by unpaired Student's *t*‐test

Foetuses from MNE developed CHDs ranging from atrial and ventricular sepal defects (ASD and VSD), double outlet right ventricle (DORV), hypoplastic left ventricle, unguarded tricuspid orifice, trabeculation defect, thickened pulmonary and aortic valves to coronary artery malformation (Table 1). MNE dose‐dependently increased the incidence of CHDs in foetuses from 0% in controls to 13% and 33% in 0.75 and 1.5 mg/kg/day nicotine treatments, respectively (*p* < 0.001). Figure 2A–J shows the typical examples of ostium secundum ASD, perimembranous septum VSD, thickened pulmonary and aortic valves, and LV hypertrophy in the MNE (1.5 mg/kg/day) group as well as corresponding normal controls. An E18.5 foetus with MNE had DORV in which both the pulmonary artery and aorta were connected to the RV (Figure K and L). Figure 2M–N shows an unguarded tricuspid orifice^33^ with ventricular hypertrophy and no trabeculation in the RV in a P0 heart with MNE (1.5 mg/kg/day). Figure 2O–P shows a vestibular ASD,^34^ and a hypoplastic heart with ostium secundum ASD, aortic stenosis and hypoplastic left ventricle in the MNE (0.75 mg/kg/day) group. Both pulmonary artery orifice diameter and aortic orifice area were significantly smaller while pulmonary valve thickness and the aortic valve to total aortic orifice area ratio were significantly higher with MNE (1.5 mg/kg/day) compared to the controls (Figure 2Q–T), indicating that outflow tract and semilunar valve remodelling^35^ are negatively impacted by MNE at 1.5 mg/kg/day. No apparent abnormalities of outflow tract and semilunar valves were seen with MNE 0.75 mg/kg/day except one mouse that had aortic stenosis associated with hypoplastic LV (Figure 2P).

**TABLE 1:** The rate of congenital heart defects in the control and MNE offspring

	Control		MNE			
Nicotine (mg/kg/day)	0		0.75		1.5	
Congenital Heart Defects						
n/litters	31/5		24/3		61/8	
	*n*	%	*n*	%	*n*	%
Normal	31	100	21	87	38	67
Abnormal	0	0	3	13	20	33***
Atrial septal defect	0	0	2	8	4	13*
Ventricular septal defect	0	0	0	0	1	2
Double outlet right ventricle	0	0	0	0	1	2
Hypoplastic left heart	0	0	1	4	0	0
Unguarded tricuspid orifice	0	0	0	0	2	3
Thickened pulmonary valve	0	0	0	0	5	8
Thickened aortic valve	0	0	0	0	5	8
RV hypertrophy	0	0	1	4	5	8
LV hypertrophy	0	0	2	8	11	18**
Trabeculation defect	0	0	1	4	8	13*
Coronary Artery Defects						
n/litters	15/5		24/3		61/8	
	*n*	%	*n*	%	*n*	%
Normal	15	100	23	96	43	70
Abnormal	0	0	1	4	18	30***

Note

Foetuses were examined at E17.5 to P0. n/litters indicate number of foetuses and litters, respectively.

Abbreviations: LV, left ventricle; RV, right ventricle.

*p < 0.05, **p < 0.01, ***p < 0.001 vs. untreated control by chi‐squared test.

### 3.3 MNE induces hypoplastic coronary arteries in mice

**FIGURE 3:**
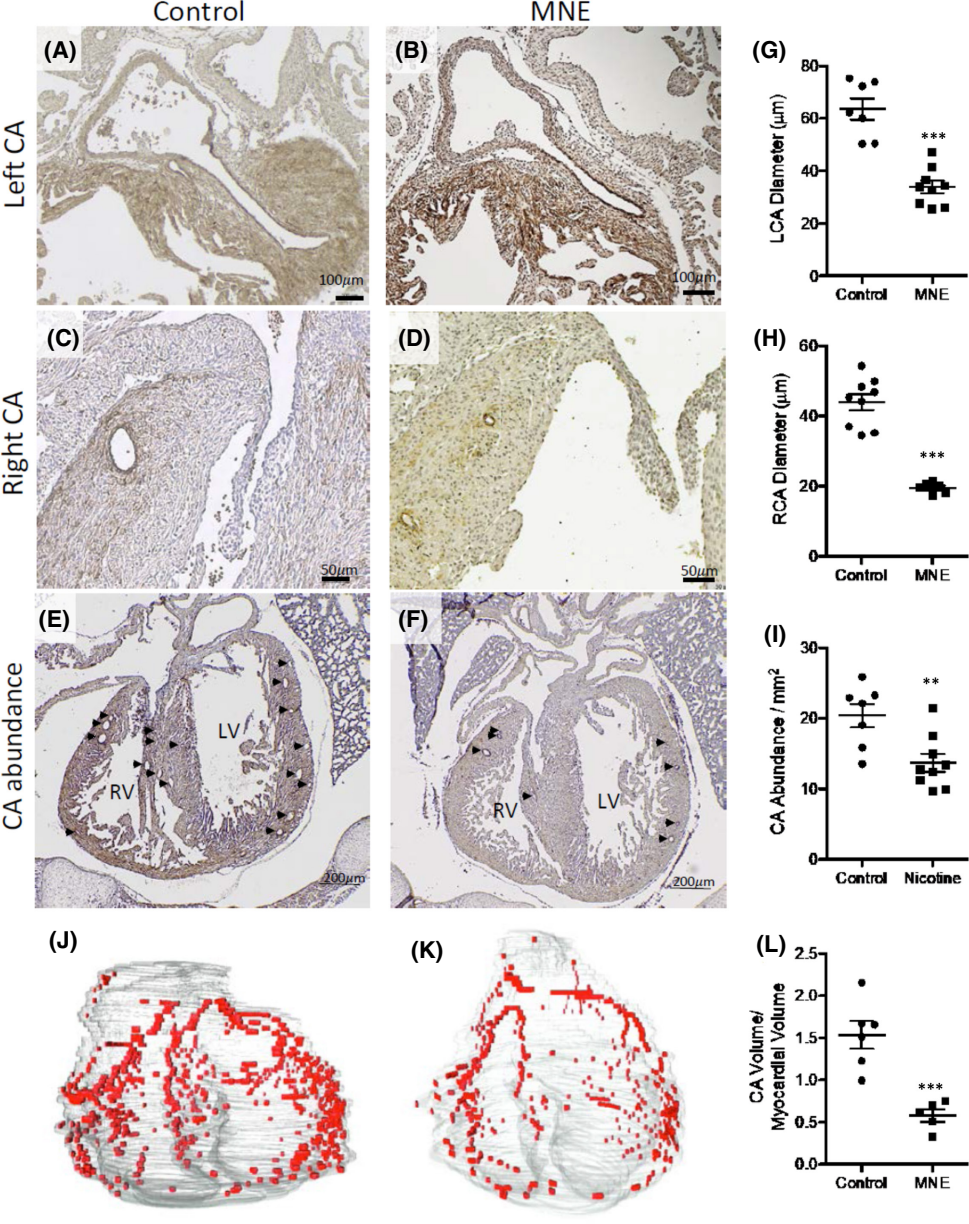
Coronary artery malformation in foetuses of MNE (1.5 mg/kg/day). A, C and E are coronary arteries in control foetuses. B, D and F are coronary arteries of foetuses with MNE. Arrows point to coronary artery openings in E and F. Quantification of left and right coronary artery diameter, and abundance (G, H and I, respectively). J and K are examples of 3D reconstructions of coronary arteries in control and MNE foetuses, respectively. The volume of coronary arteries was significantly lower in MNE foetuses (L). All images are from E18.5 hearts. **p < 0.01, ***p < 0.001 vs. untreated control by unpaired Student's *t*‐test. LCA, left coronary artery; RCA, right coronary artery; CA, coronary artery; LV, left ventricle; RV, right ventricle

Coronary arteries were identified on E18.5 heart sections by α‐smooth muscle actin immunostaining. Representative images of the coronary arteries, and their abundance on 4‐chamber view heart sections are shown in Figure 3A–F. MNE (1.5 mg/kg/day) foetuses had smaller diameters of left and right coronary arteries, and their abundance was significantly lower (Figure 3G–I). 3D reconstructions of the heart showed that the volume of the coronary artery tree was significantly lower in MNE foetuses (Figure 3J–L). NME at 0.75 and 1.5 mg/kg/day induced 4% and 30% of coronary artery malformation in foetuses, respectively (Table 1). Foetuses with both CHD and coronary artery malformation were 4% and 16% in 0.75 and 1.5 mg/kg/day MNE, respectively.

### 3.4 MNE changes gene expression in foetal hearts

To study the effects of MNE (1.5 mg/kg/day) on the expression of genes critical to embryonic heart development, foetal hearts were collected at E10.5 and RT‐qPCR analysis was performed. Changes in gene expression on pathways responsible for angiogenesis, cell proliferation, differentiation and EMT are summarized in Table 2. The mRNA levels of *Hif1*α, *Bmp10*, *ALDH1a2*, *bFGF*, *Snail1*, *Slug*, *TGF*‐*β1*, *Notch1*, *eNOS*, *CyclinD1* and β‐*MHC* were significantly lower in MNE hearts in comparison with the controls (*p* < 0.05), while no significant changes were found in *Tbx18*, *Gata4*, *Nkx2*.*5*, *PKCi* or *Tbx5* mRNA levels (Table 2).

**TABLE 2:** Gene expression of transcription and growth factors critical to heart and coronary morphogenesis in E10.5 hearts and hypertrophic growth factors in E18.5 hearts of fetuses from MNE (1.5 mg/kg/day) and control groups

	Control	MNE
E10.5		
Hif1α	0.00219 ± 0.00032	0.00134 ± 0.00018*
Bmp10	0.00925 ± 0.00108	0.00359 ± 0.00042***
ALDH1a2	0.02157 ± 0.00308	0.00997 ± 0.00238*
bFGF	0.00431 ± 0.00053	0.00240 ± 0.00033*
Snail1	0.00069 ± 0.00012	0.00036 ± 0.00005*
Slug	0.00050 ± 0.00008	0.00022 ± 0.00005*
TGF‐β1	0.01648 ± 0.00228	0.01037 ± 0.00121*
Notch1	0.00035 ± 0.00003	0.00019 ± 0.00002*
eNOS	0.00606 ± 0.00091	0.00336 ± 0.00031*
Tbx18	0.00080 ± 0.00011	0.00061 ± 0.00012
Cyclin D1	0.00213 ± 0.00036	0.00127 ± 0.00016*
β‐MHC	0.04210 ± 0.00614	0.01688 ± 0.00305**
Gata4	0.02638 ± 0.00509	0.01977 ± 0.00363
Nkx2.5	0.00357 ± 0.00059	0.00322 ± 0.00053
PKCi	0.00315 ± 0.00032	0.00296 ± 0.00067
Tbx5	0.00327 ± 0.00069	0.00209 ± 0.00031
E18.5		
TGFβ1	0.01179 ± 0.00071	0.01506 ± 0.00109*
Cyclin D1	0.00982 ± 0.00079	0.01384 ± 0.00117*
β‐MHC	1.03500 ± 0.09925	1.45000 ± 0.12770*
BNP	0.02433 ± 0.00223	0.03266 ± 0.00256*

Note

RT‐qPCR analysis of mRNA levels in relation to 28S ribosomal RNA in E10.5 and E18.5 ventricular myocardium. Data are mean ±SEM.

* *p* < 0.05 **p < 0.01, ***p < 0.001 vs. untreated control by unpaired Student's *t*‐test, *n* = 7–8 per group.

To determine signalling pathways on cardiac hypertrophy during later stages of heart development, gene expression was assessed in E18.5 hearts using RT‐qPCR analysis. The levels *TGF*‐*β1*, *CyclinD1*, β‐*MHC* and *BNP* mRNAs were significantly higher in MNE hearts (1.5 mg/kg/day) in comparison with the controls (*p* < 0.05, Table 2).

### 3.5 MNE lowers cell proliferation

**FIGURE 4:**
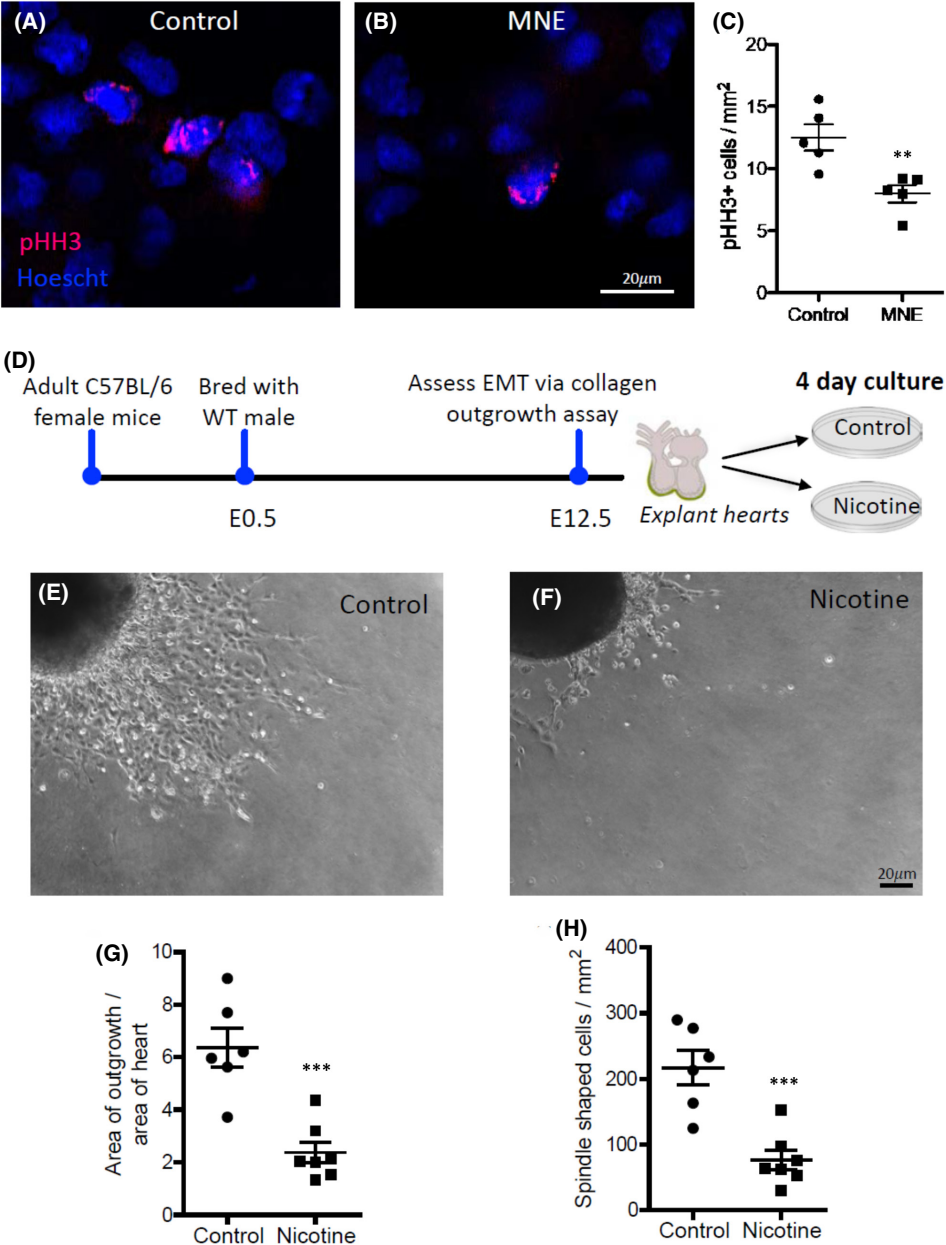
Effects of nicotine on cell proliferation and epicardial EMT. A‐C, MNE (1.5 mg/kg/day) inhibits cell proliferation of foetal hearts in vivo. A and B are representative images of phospho‐histone H3 (pHH3) immunostaining of heart sections from control and MNE foetuses at E10.5, respectively. C, Quantification of pHH3‐positive cells per mm^2^ in E10.5 hearts. D‐H, Nicotine decreases epicardial EMT ex vivo. D‐F, E12.5 heart explants were seeded on collagen coated wells in the presence or absence of nicotine (100 ng/ml). Epicardial cells migrated from the heart explant and underwent EMT to become spindle‐shaped, mesenchymal like cells. Four days after nicotine incubation, cell outgrowth area (G) and spindle‐shaped cell numbers (H) were quantified. **p < 0.01, ***p < 0.001 compared to controls by unpaired Student's *t*‐test

Cell proliferation is critical to heart morphogenesis and coronary artery development. E10.5 hearts were immunostained with the phosphorylated histone H3 (pHH3), a biomarker of proliferating cells (Figure 4A,B). The pHH3 positive cells were quantified on 4–5 sections per heart. The number of pHH3 positive cells was significantly lower in the ventricular myocardium of the MNE (1.5 mg/kg/day) group in comparison with the control group (*p* < 0.01, Figure 4C).

### 3.6 Nicotine exposure inhibits epicardial EMT *ex vivo*

Epicardial EMT is essential to coronary artery development. To study the effect of nicotine on epicardial EMT, E12.5 heart explants were seeded on collagen coated wells to allow epicardial cell outgrowth and EMT to become spindle‐shaped, mesenchymal like cells (Figure 4D–F). The area of outgrowth and the number of spindle‐shaped cells were quantified and normalized to heart explant area (Figure 4G,H). Nicotine‐treated heart explants showed significantly less outgrowth and fewer spindled‐shaped cells in comparison with the controls (*p* < 0.05, Figure 4G,H). These data suggest that nicotine exposure inhibits epicardial EMT of the foetal heart.

### 3.7 MNE increases oxidative stress in foetal hearts

**FIGURE 5:**
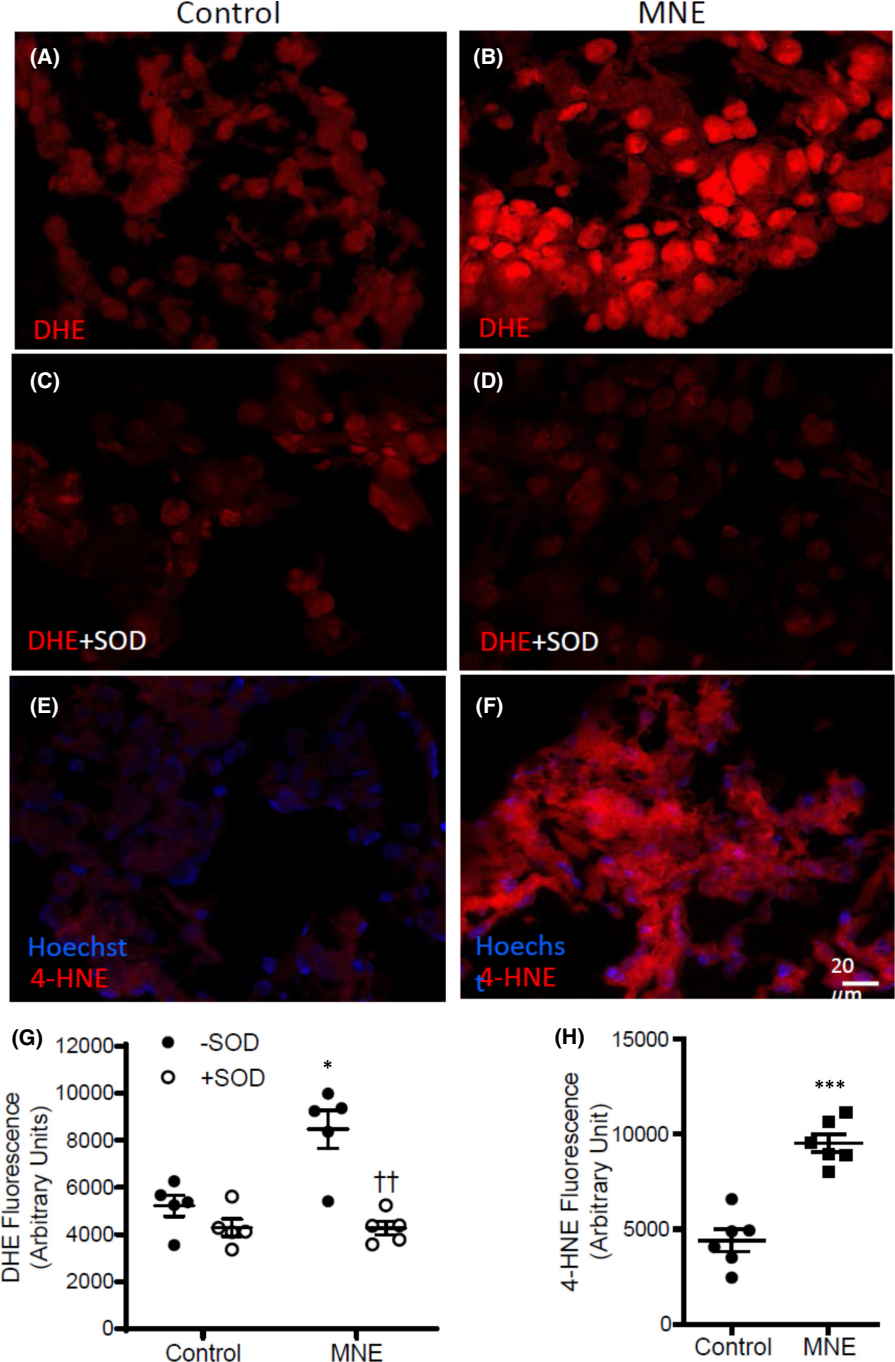
Effects of MNE (1.5 mg/kg/day) on oxidative stress. A and B are representative images of dihydroethidium (DHE) stained heart sections to assess superoxide levels in control and MNE foetuses at E10.5, respectively. Tissues were incubated with superoxide dismutase (SOD) to verify superoxide specific signals in control (C) and MNE (D) foetal heart sections. G, the intensity of DHE fluorescence was quantified by densitometry. *p < 0.05 vs control, ††*p* < 0.01 vs MNE without SOD using two‐way ANOVA followed by Tukey's multiple comparisons test. E and F, E10.5 heart sections were immunostained using an anti‐4‐hydroxynonenal (4‐HNE) antibody to assess lipid peroxidation in control and MNE foetal hearts. H, the intensity of 4‐HNE fluorescence was quantified by densitometry. ***p < 0.001 vs control by unpaired Student's *t*‐test

To assess the effect of MNE on oxidative stress in foetal hearts, superoxide levels were determined in E10.5 hearts using dihydroethidine (DHE) as a probe. To verify that the DHE fluorescence was superoxide, some heart sections were incubated with a superoxide scavenger, superoxide dismutase (SOD). Fluorescence images were taken on 5 sections per heart for all groups at the same exposure, and intensity of the fluorescence was quantified using densitometry (Figure 5A–D). The results showed that hearts with MNE (1.5 mg/kg/day) had significantly higher superoxide levels than the control group (*p* < 0.05), which was attenuated by SOD (*p* < 0.001, Figure 5G).

To assess oxidative damage, levels of 4‐hydroxynonenal, a product of lipid peroxidation were determined in E10.5 hearts using immunostaining. Fluorescence images were taken at the same exposure on 5 sections per heart in control and NME (1.5 mg/kg/day) groups (Figure 5E,F). Our data showed that the MNE hearts had significantly higher levels of lipid peroxidation in comparison to the control group (*p* < 0.001, Figure 5H).

## 4 DISCUSSION

Defining safety guidelines of substance use during pregnancy is a critical focus in preventative healthcare to lower the incidence of congenital defects. A risk factor such as MNE could be controlled by establishing updated guidelines for NRT use during pregnancy. Therefore, we evaluated MNE’s effects on heart development and underlying pathways. MNE dose‐dependently induced both CHDs and altered coronary artery development in the foetuses of mice. MNE resulted in higher ROS levels in developing hearts. High ROS levels contribute to CHDs and malformation of coronary arteries in mouse offspring.^36^ We established for the first time a model of CHDs induced by MNE in mice.

To determine CHD pathogenesis and coronary artery defects, it is vital to determine mechanisms underlying MNE’s effects on foetal hearts during crucial developmental stages. Our data provide evidence that MNE impairs cell proliferation and epicardial EMT during embryonic development. In E10.5 hearts, when cardiac septation, chamber valve formation and coronary artery genesis begins. Less cell proliferation was found in foetuses of MNE than those of the control mice. Also, fewer pHH3‐stained cells in these hearts indicate less proliferation than those of control hearts. Mechanisms of lower proliferation with MNE were indicated by gene expression analysis. Foetal hearts of MNE mice had significantly lower expressions of *CyclinD1*, *bFGF*, *Bmp10* and β‐*MHC*, markers of cardiomyocyte growth and proliferation.

Notably, the mRNA level of *Hif1*α, an important controller of epicardial EMT, including the myocardial migration of EPDCs,^37^ was lower in the MNE mouse foetuses than those of control foetuses. ROSs alter *Hif1α* expression. Lower ROS levels cause higher *Hif1α* expression, which promotes cardiovascular differentiation. On the other hand, higher levels of ROS inhibit *Hif1α expression*.^38^ Other important genes for EMT include *Snail1*, *Slug*, *bFGF*, *eNOS*, *Notch1* and *ALDH1a2*, their mRNA levels were all lower with MNE. *Snail1*, *Slug* and *ALDH1a2* are all downstream of *Hif1α*, while *Notch1* activates the expression of *Snail1*, *Slug* and *TGFβs*.^39^ *ALDH1a2* promotes retinoic acid signalling that is essential for the formation of the epicardium, its attachment to the myocardium, subsequent growth and proliferation, and the development of the coronary arteries.^39^, ^40^ *bFGF* mediates retinoic acid signalling, in embryonic hearts, promoting EMT and vasculogenesis. In our study, MNE lowers *bFGF* expression and impairs signalling of *Snail1*/*Slug* and *ALDH1a2*/*bFGF*, which will result in lower epicardial EMT and coronary artery malformations in foetal hearts.

Lower cell proliferation at E10.5 does not explain the ventricular hypertrophy in many MNE E18.5 foetuses in our study. To gain insights into underlying molecular mechanisms, expression of various hypertrophic genes was evaluated in E18.5 foetal hearts. *TGF*‐*β1*, β‐*MHC*, *BNP* and *Cyclin D1* had higher expressions in MNE hearts than those of control hearts. *In utero* cardiac hypertrophy is due to cardiomyocyte proliferation and hypertrophy.^41^ *Cyclin D1* has important roles for proliferation and hypertrophic regulation.^42^ Increased ventricular *β1* are associated with cardiac hypertrophy.^43^, ^44^ Upregulation of β‐*MHC* is a marker of cardiac hypertrophy.^45^ Our results in mice support the conclusion that MNE causes ventricular hypertrophy. Additionally, haemodynamic expressions of *BNP* and *TGF*‐changes due to thickened semilunar valves, narrowed orifice of the aorta and pulmonary artery, and septal defects may also contribute to ventricular hypertrophy in MNE hearts.

Excessive production of ROS has been shown to be detrimental to embryogenesis, and oxidative stress promotes the development of CHDs.^26^, ^36^ In our study, MNE hearts at E10.5 had higher superoxide and lipid peroxidation levels, indicating oxidative damage.^46^ Nicotine binding to nicotinic acetylcholine receptors results in increased ion influx, which increases intracellular Ca^2+^ and subsequently disturbs intracellular signalling and organelle function.^47^ Increased intracellular Ca^2+^ in the foetus causes mitochondrial production of ROS production.^48^ Increased mitochondrial ROS production disrupts the endogenous antioxidant capacity which can lower cell proliferation.^36^ During embryonic development, high ROS produces DNA damage, and oxidation of proteins and lipids, as well as affecting cellular apoptosis, proliferation, differentiation and inflammation.^36^ Increased ROS also reduces nitric oxide bioavailability and eNOS coupling.^30^ eNOS deficiency leads to CHDs and malformations of the coronary arteries.^49^, ^50^ Also, ROS regulates vasculogenesis.^51^ Physiological levels of ROS promote normal vessel development; however, excessive ROS during cardiac development inhibits vessel development.^36^ Our results support that higher levels of ROS contribute to CHDs and coronary artery malformation in MNE mouse offspring.

Dose‐dependent higher CHDs in offspring of MNE indicate nicotine levels enhance cardiac malformations. A cigarette contains 0.8–1.9 mg of nicotine on average and typically results in 10–50 ng/ml peak plasma levels in humans with a plasma half‐life of less than 20 min and a distribution volume 2.6 times body weight, indicating nicotine quickly gets into the tissues after smoking.^28^, ^29^ Plasma half‐life of nicotine is less than 7 min in mice.^52^ A limitation of our study is that we were unsuccessful in measuring plasma nicotine levels in pregnant mice. Thus, we are unable to correlate plasma nicotine levels with CHDs. Also, we did not implant osmotic pumps in control mice, which may interfere with pregnancy. However, female mice were bred 14 days after pump implantation. Thus, any adverse effect of the surgery or pump on pregnancy would be minimal.

In conclusion, in mice, MNE dose‐dependently induces CHDs and malformations of the coronary arteries. In embryonic hearts, excess ROS levels induced by MNE alter genetic expression profiles, cellular proliferation and EMT, leading to the development of CHDs and coronary artery malformation. These findings elucidate the adverse effects of nicotine use in women during pregnancy. Our model provides a future opportunity to further dissect mechanisms of MNE‐induced CHDs.

## CONFLICTS OF INTEREST

The authors confirm that there are no conflicts of interest.

## AUTHOR CONTRIBUTION

**Elizabeth R. Greco:** Conceptualization (supporting); Data curation (equal); Formal analysis (equal); Investigation (equal); Methodology (equal); Writing – original draft (equal); Writing – review & editing (equal). **Anish Engineer:** Conceptualization (equal); Data curation (equal); Formal analysis (equal); Investigation (equal); Methodology (equal); Writing – review & editing (equal). **Tana Saiyin:** Conceptualization (equal); Data curation (equal); Formal analysis (equal); Investigation (equal); Methodology (equal); Writing – review & editing (equal). **Xiangru Lu:** Conceptualization (equal); Data curation (supporting); Investigation (equal); Methodology (equal); Writing – review & editing (equal). **MengQi Zhang:** Conceptualization (supporting); Data curation (equal); Formal analysis (equal); Investigation (equal); Methodology (equal); Writing – review & editing (equal). **Douglas L. Jones:** Conceptualization (equal); Supervision (equal); Writing – review & editing (equal). **Qingping Feng:** Conceptualization (lead); Funding acquisition (lead); Investigation (equal); Project administration (lead); Writing – original draft (equal); Writing – review & editing (lead).

## Supporting information

Table S1

## Data Availability

The data that support the findings of this study are available from the corresponding author upon reasonable request.
