# Supplementary material for: Maternal nicotine exposure induces congenital heart defects in the offspring of mice
Source: J Cell Mol Med. 2022 May 6;26(11):3223–34. doi: 10.1111/jcmm.17328 (PMC9170818; doi:10.1111/jcmm.17328)
Supplement: Supplementary file 1 — Table S1 [file JCMM-26-3223-s001.pdf]

**Maternal Nicotine Exposure Induces Congenital Heart Defects in the Offspring of Mice**

Elizabeth R. Greco, Anish Engineer, Tana Saiyin, Xiangru Lu, MengQi Zhang, Douglas L. Jones, Qingping Feng

**Supplementary Table 1. Specific primer sequences for real-time PCR analysis**

| Gene | Accession No. | Product Size | $T_M$ (°C) | Primer Sequence |
|---|---|---|---|---|
| *Nkx2.5* | NM_008700.2 | 162 | 62 | F: TCAATGCCTATGGCTACAACG<br>R: GACGCCAAAGTTCACGAAGTT |
| *Gata4* | NM_008092.3 | 134 | 64 | F: CACTATGGGCACAGCAGCTC<br>R: GCCTGCGATGTCTGAGTGAC |
| *Bmp10* | NM_009756.3 | 235 | 62 | F: CCACTCGGATCAGGAGGAAC<br>R: CACACAGCAGGCTTTGGAAG |
| *eNOS* | NM_008713.4 | 165 | 64 | F: ATTTCCTGTCCCCTGCCTTCC<br>R: ATGGTTGCCTTCACACGCTTC |
| *Notch1* | NM_008714.3 | 142 | 62 | F: TAACGGAGTACGGCCATGT<br>R: CAGCTTGCACAACCAGACAGA |
| *Cyclin D1* | NM_007631.2 | 135 | 62 | F: CTGACACCAATCTCCTCAACG<br>R: CTCACAGACCTCCAGCATCCA |
| *Hif-1α* | NM_010137.3 | 159 | 63 | F: CTTGGACGCTCTGCCTATGA<br>R: AGGTTGCGGGGGTTGTAGAT |
| *bFGF* | NM_008006.2 | 174 | 64 | F: CAAGGGAGTGTGTGCCAACC<br>R: TGCCCAGTTCGTTTCAGTGC |
| *Tbx5* | NM_011537.3 | 103 | 63 | F: AGGAGCACAGTGAGGCACAA<br>R: GGGCCAGAGACACCATTCTC |
| *Tbx18* | NM_023814.4 | 199 | 64 | F: GAGCAGCAACCCGTCTGTGA<br>R: GGGACTGTGCAATCGGAAGG |
| *Snail1* | NM_011427.2 | 114 | 54 | F: CACACGCTGCCTTGTGTCT<br>R: GGTCAGCAAAAGCACGGTT |
| *Slug* | NM_011415 | 161 | 67 | F: CAACGCCTCCAAGAAGCCCA<br>R: GAGCTGCCGACGATGTCCAT |
| *ALDH1a2* | NM_009022.4 | 219 | 64 | F: GGCAGCAATCGCTTCTCACA<br>R: CAGCACTGGCCTTGGTTGAA |
| *TGF-β1* | NM_011577.1 | 120 | 61 | F: GCCCGAAGCGGACTACTATG<br>R: CACTGCTTCCCGAATGTCTG |
| *PKCi* | XM_006535411 | 220 | 60 | F: TATGGCTTCAGCGTTGACTG<br>R: CCTTTGGGTCCTTGTTGAGA |
| *β*-MHC | NM_08072 | 204 | 63 | F: ACACACGAGCATGTGAATAG<br>R: GTGGGTAAGGGAGTGGGG |
| *BNP* | NM_008726 | 189 | 60 | F: TGGGAATTAGCCATGTGAGAG<br>R: TTTGGGTGTTCTTTTGTGAGG |
| SX | AC165339.2 | 282 | 62 | F: GATGATTTGAGTGGAAATGTGAGGTA<br>R: CTTATGTTTATAGGCATGCACCATGTA |
| 28S | NR_003279.1 | 178 | 61 | F: GGGCCACTTTTGGTAAGCAG<br>R: TTGATTCGGCAGGTGAGTTG |

F: Forward primer, R: reverse primer
